# DNA Damage Sensing and TP53 Function as Modulators of Sensitivity to Calicheamicin-Based Antibody–Drug Conjugates for Acute Leukemia

**DOI:** 10.3390/cancers18010067

**Published:** 2025-12-25

**Authors:** Camryn M. Pettenger-Willey, George S. Laszlo, Margery Gang, Frances M. Cole, Colin D. Godwin, Sarah Erraiss, Pritha Chanana, Allie R. Kehret, Junyang Li, Jacob W. Barton, Meghann M. Yochim, Eduardo Rodríguez-Arbolí, Roland B. Walter

**Affiliations:** 1 Translational Science and Therapeutics Division, Fred Hutchinson Cancer Center, Seattle, WA 98109, USA; 2 Hematology/Oncology Fellowship Program, Fred Hutchinson Cancer Center/University of Washington, Seattle, WA 98109, USA; 3 Shared Resources, Fred Hutchinson Cancer Center, Seattle, WA 98109, USA; 4 Department of Hematology, Hospital Universitario Virgen Del Rocío, Instituto de Biomedicina de Sevilla (IBIS/CSIC), University of Seville, 41013 Seville, Spain; 5 Department of Medicine, Division of Hematology and Oncology, University of Washington, Seattle, WA 98195, USA; 6 Department of Laboratory Medicine and Pathology, University of Washington, Seattle, WA 98195, USA

**Keywords:** acute leukemia, calicheamicin, CD22, CD33, CRISPR/Cas9, drug screen, gemtuzumab ozogamicin, inotuzumab ozogamicin

## Abstract

Background/Objectives: Approved for treatment of acute leukemia, gemtuzumab ozogamicin (GO) and inotuzumab ozogamicin (InO) are antibody–drug conjugates (ADCs) that deliver a toxic calicheamicin (CLM) derivative. The resistance mechanisms to GO/InO remain incompletely understood. Methods: We performed a genome-wide clustered regularly interspaced short palindromic repeat (CRISPR)/Cas9 screen for CLM sensitivity genes, and then performed confirmatory cytotoxicity assays. Results: Several DNA damage pathway regulation genes were identified, most notably *TP53*. Across 13 acute leukemia cell lines, the six *TP53*-mutant cell lines (*TP53*^MUT^) were indeed 10- to 1000-fold less sensitive to CLM than the seven *TP53*^WT^ cell lines. In five *TP53*^WT/KO^ syngeneic cell line pairs we generated, *TP53*^KO^ cells were significantly less sensitive to CLM than their *TP53*^WT^ counterparts. In *TP53*^WT^ but not *TP53*^MUT^ cells, the MDM2 inhibitor and p53 activator, idasanutlin, enhanced CLM cytotoxicity, demonstrating that decoupling of cells from MDM2-p53 regulation sensitizes leukemia cells to CLM. The ATM inhibitors AZD1390 and lartesertib also significantly enhanced CLM efficacy but did so independent of the *TP53* status. In contrast, neither an ATR inhibitor, Chk1/Chk2 inhibitor, Chk2 inhibitor, or a PARP inhibitor significantly impacted CLM-induced cytotoxicity across the thirteen cell lines. Together, our studies identify ATM, MDM2, and *TP53*—which are in the same cellular response to DNA damage pathway—as key modulators of CLM-induced cytotoxicity in acute leukemia cells. Conclusions: These results support further evaluation of combination therapies with corresponding small-molecule inhibitors (currently pursued for therapy of other cancers) toward clinical testing as novel strategies to increase the efficacy of CLM-based ADCs such as GO and InO.

## Simple Summary

Acute leukemias are difficult-to-treat blood cancers which many patients will eventually die from despite intensive chemotherapies and bone marrow transplantation. Antibodies that recognize proteins on leukemia cells and deliver a cell toxin (so-called antibody–drug conjugates) have been developed to improve these outcomes. Two such drugs, gemtuzumab ozogamicin (GO, for acute myeloid leukemia) and inotuzumab ozogamicin (IO, for acute lymphoblastic leukemia), have been approved for use in patients but are not always effective. In our laboratory research, we undertook genome-wide screening to discover genes that are associated with response or resistance to the toxin (calicheamicin) that both GO and IO contain. Our studies revealed the importance of several DNA damage pathway regulation genes for the anti-leukemia activity of calicheamicin, including *TP53*, *ATM*, and *MDM2*. Building on these data, we then identified several small-molecule inhibitors that increased GO/IO efficacy—findings that support further evaluation of these combination therapies with clinical testing.

## 1. Introduction

Acute leukemias remain difficult to treat despite multi-agent chemotherapy, allogeneic hematopoietic cell transplantation, and the increasing availability of immunotherapeutics and small-molecule inhibitors [1,2,3,4,5,6]. Antibody–drug conjugates (ADCs) have long been pursued to improve cure rates in these malignancies, with a major focus on CD33 (for acute myeloid leukemia [AML]) and CD22 (for B-acute lymphoblastic leukemia [B-ALL]). Improved outcomes with the CD33 ADC, gemtuzumab ozogamicin (GO) [7,8,9], and the CD22 ADC, inotuzumab ozogamicin (InO) [10,11,12], validate these efforts. Still, in many patients, GO and InO are insufficiently effective. Rational use of combinatorial therapies may be one strategy to widen the therapeutic reach of these ADCs in acute leukemia.

Both GO and InO deliver a derivative of calicheamicin-γ_1_^I^ (N-acetyl gamma calicheamicin-γ_1_^I^ dimethyl hydrazide [CLM]) as a toxic payload. Once internalized and released from the antibody, CLM binds DNA in the minor groove and undergoes structural changes, leading to a diradical that abstracts hydrogens from the phosphodiester backbone of DNA, resulting in single- and double-strand breaks [8,13]. G_2_/M cell cycle arrest follows DNA damage sensing, and cell death—primarily via mitochondrial apoptosis pathways—ensues if damage is overwhelming. Modulation or loss of target antigen (for CD22), DNA damage response dysregulation, drug efflux pumps, and apoptotic dysregulation have been implicated in resistance to CLM-based ADCs [8,13,14,15]. However, the cellular mechanisms allowing acute leukemia cells to resist GO and InO remain incompletely understood. Consequently, to identify genetic vulnerabilities and new biomarkers that could inform selection of patients most likely to benefit from CLM-based ADCs and to develop combination therapies to augment the efficacy of these ADCs, we performed a genome-wide clustered regularly interspaced short palindromic repeat (CRISPR)/Cas9 screen to discover genes associated with CLM sensitivity.

## 2. Materials and Methods

### 2.1. Therapeutics and Chemicals

CLM was provided by Pfizer (New York, NY, USA). AZD1390, BML-277, ceralasertib, idasanutlin, lartesertib, and prexasertib were obtained from Selleck Chemicals (Houston, TX, USA). Veliparib was obtained from Apexbio Technology (Houston, TX, USA). For drug selection, blasticidin was purchased from Research Projects International (Mount Prospect, IL, USA), puromycin was obtained from Gold Biotechnology (Saint Louis, MO, USA), and G418 was obtained from ThermoFisher Scientific (Waltham, MA, USA).

### 2.2. Parental Human Acute Leukemia Cell Lines

Human myeloid and lymphoid EOL-1, HL-60, K562, Kasumi, KG-1, MOLM-13, REH, TF-1, and THP-1 cells were grown in RPMI-1640 medium with 10% fetal bovine serum (FBS; for EOL-1, K562, REH, and THP-1 cells), 20% FBS (for Kasumi, KG-1, and MOLM-13 cells), or 10% BCS (for HL-60 cells). TF-1 cells were supplemented with 4 ng/mL GM-CSF (Peprotech; Cranbury, NJ, USA). Human myeloid and lymphoid OCI-AML3 and RS4;11 cells were grown in alpha-MEM with 10% FBS. ML-1 and MV4;11 cells were grown in IMDM and 10% FBS, with MV4;11 cells supplemented with 5 ng/mL GM-CSF with 1x Insulin–Transferrin–Selenium supplement (ThermoFisher Scientific). All cell lines were grown with penicillin/streptomycin, tested for mycoplasma contamination (MycoAlert Mycoplasma Detection Kit; Lonza, Basel, Switzerland), and authenticated using standard STR CODIS typing.

### 2.3. Lentiviral Expression Vectors

Lentiviral vectors containing Cas9 expression cassettes, LentiCas9-Blast (plasmid ID # 52962), Lenti-iCas9-neo (plasmid ID # 85400), lentiCRISPR v2 (plasmid ID # 52961), the lentiGuide-puro (plasmid ID # 52963) for expression of individual gRNAs, and the Brunello CRISPR knockout pooled library lentiviral particle prep with puromycin selection cassette, including 76,441 unique gRNAs covering over 19,000 genes, each gene targeted with 4 unique sgRNAs (ID # 73178-LV), were all purchased from Addgene (Watertown, MA, USA). The CD33 knockout (KO) sgRNA targeting exon 1 of CD33 (sequence 5′-CTGCTGCCCCTGCTGTGGGC-3′) was cloned into lentiGuide-puro with standard cloning techniques and confirmed by Sanger sequencing. Cas9 expression lentiviral vector particles and CD33 sgRNA lentiviral particles were prepared as described [16]. Lentivirally transduced sublines, when viral titering was possible, were generated at multiplicities of infection (MOI) of 0.25–25. EGFP-positive cells were isolated by fluorescence-activated cell sorting (FACS) and re-cultured for further analysis/use.

### 2.4. CRISPR/Cas9 Whole-Genome-Wide Screening

ML-1, K562 or OCI-AML3 cell lines were transduced with viral particles encoding LentiCas9-Blast [17] and selected with blasticidin to generate cells with constitutive Cas9 expression. To reduce the frequency of cells harboring > 1 sgRNA, cells were transduced with MOI of 0.3. Viral transduction of Brunello whole-genome targeting CRISPR/Cas9 library was performed at ~150–300x coverage. Two days later, transduced cells were selected with puromycin. Subsequently, cells were expanded to allow for treatment with either CLM or vehicle. CLM was administered every 3 days at a concentration aimed at inducing 15–30% cell death. Samples were collected on days 0, 6, and 12. Genomic DNA was purified and embedded sgRNA was amplified and barcoded by PCR, sequenced, and analyzed to determine sgRNAs under- or over-represented in CLM-treated populations compared to controls. Bowtie was used for alignment and guide quantification. Model-based Analysis of Genome-wide CRISPR-Cas9 Knockout (MAGeCK) was used in conjunction with a modified robust ranking aggregation (RRA) algorithm to identify positive/negative selected genes across the two conditions, and hits with a false discovery rate (FDR) < 5% were considered for further study.

### 2.5. Quantification of CD33 Expression

CD33 cell surface expression was quantified with a PE-conjugated CD33 antibody (clone p67.6; BD Biosciences, Franklin Lakes, NJ, USA) and reported as median fluorescence intensity (MFI).

### 2.6. TP53 Genomic Analysis

We used 500 ng of purified genomic DNA from human acute leukemia cells to assess the *TP53* allelic status as described [18].

### 2.7. Quantification of Drug-Induced Cytotoxicity

Human acute leukemia cells were incubated in 96-well round-bottom plates at 5–10 × 10^3^ cells/well with various concentrations of CLM and/or AZD1390, BML-277, ceralasertib, idasanutlin, lartesertib, prexasertib, or veliparib. After 3 days, cell numbers and drug-induced cytotoxicity, using 4′,6-diamidino-2-phenylindole (DAPI) to detect non-viable cells, were determined flow cytometrically.

### 2.8. Generation of Human Leukemia Cell Lines with Deletion of TP53

Leukemia cell sublines with deletion of *TP53* were generated as described [18]. Briefly, CRISPR/Cas9 editing was carried out by electroporating purified Cas9 protein (TrueCut Cas9 V2; ThermoFisher Scientific) complexed with a pool of synthetic guide RNAs (sgRNAs) targeting *TP53* (sequences: 5′-CGCUAUCUGAGCAGCGCUCA-3′, 5′-GUGCUGUGACUGCUUGUAGA-3′, 5′-CAACAAGAUGUUUUGCCACC-3′; Synthego, Redwood City, CA, USA), using the ECM 380 Square Wave Electroporation system (Harvard Apparatus; Cambridge, MA, USA). *TP53* knockout (*TP53*^KO^) cells were selected in culture with either 1 µM (myeloid cell lines) or 2.5 µM (lymphoid cell lines) idasanutlin, which was freshly added every 2–3 days. Cytotoxicity assays were performed 14–21 days after electroporation.

### 2.9. Statistical Considerations

Statistical analyses were performed with Prism (Graphpad; La Jolla, CA, USA).

## 3. Results

### 3.1. CRISPR/Cas9 Whole-Genome Screening Identifies TP53 as a CLM Sensitivity Gene

**Figure 1:**
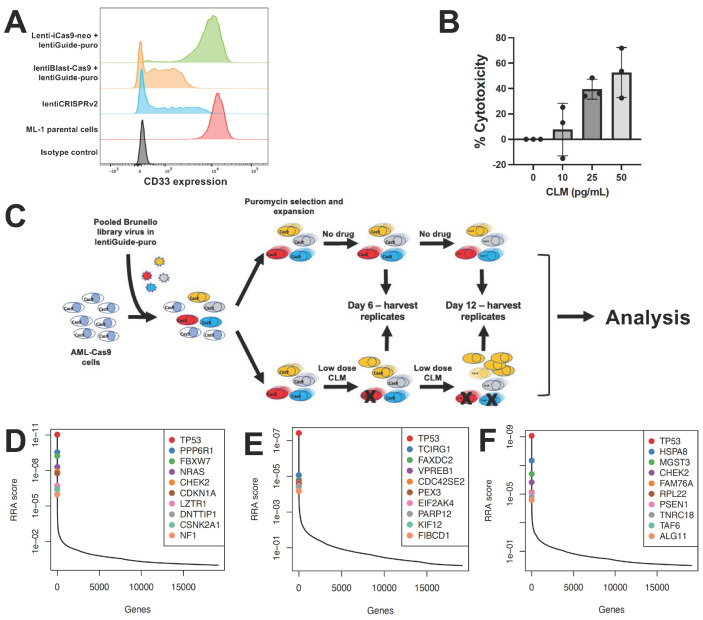
CRISPR/Cas9 whole-genome drug screening. (**A**) Optimization of CD33 gene editing. CD33 expression levels were compared in parental ML-1 cells and sublines virally transduced with sgRNA targeting CD33 and Cas9. The lentiCRISPRv2 construct is included as a positive control, and PE-conjugated isotype negative control antibody is shown. (**B**) CLM dose finding. CLM-induced cytotoxicity was assessed in ML-1 cells after 3 days. Data are presented as the mean ± SEM from 3 independent experiments performed in duplicate wells. (**C**) Schematic of CRISPR/Cas9 whole-genome sgRNA screen. (**D**–**F**) Top 10 CRISPR/Cas9 screening hits as determined by MaGeCK and RRA analysis in (**D**) ML-1, (**E**) K562, and (**F**) OCI-AML3 cells.

CRISPR whole-genome screening requires library recipient cells expressing Cas9. To identify an optimal screening platform, we compared conditional with constitutive Cas9 expression using a single guide RNA (sgRNA) to disrupt CD33 in ML-1 cells. In our studies, we contrasted an inducible Cas9 expression construct, zeocin-inducible Lenti-iCas9-neo, with constitutive Cas9 expression from the EF1a promoter-driven lentiBlast-Cas9 construct and an all-in-one single expression plasmid construct as control, lentiCRISPRv2, which combines constitutive expression of both Cas9 and sgRNA from one plasmid. While the inducible Cas9 system generated only a small subpopulation of CD33-negative cells, constitutive Cas9 expression yielded a robust CD33^KO^ phenotype (Figure 1A). Based on these results, ML-1 cells with lentiBlast-Cas9 were selected for transduction of the Brunello sgRNA library. Transduced cells were expanded under puromycin selection, and aliquots were simultaneously subjected to CLM titration to identify 25 ng/mL as the CLM dose inducing 15–30% cell death, the desired target cytotoxicity for the drug screen (Figure 1B). The screen was conducted by incubating human acute leukemia cells (ML-1, K562, OCI-AML3) in the presence or absence of CLM. Samples were collected at time 0 as well as after 6 and 12 days of culture. At these time points, cells aliquots were collected, sgRNAs sequenced, and bioinformatically analyzed (Figure 1C). As depicted in Figure 1D, this screen identified several DNA damage pathway regulation genes in ML-1 cells, including *TP53* (as the highest ranked hit overall), *PPP6R1*, *FBXW7*, *CHEK2*, *CDKN1A*, *DNTTIP1*, and *CSNK2A1* as conferring sensitivity to CLM. These findings were confirmed in K562 cells (for *TP53*; Figure 1E) and OCI-AML3 cells (for *TP53* and *CHEK2*; Figure 1F).

### 3.2. Validation of TP53 as a CLM Sensitivity Gene

**Figure 2:**
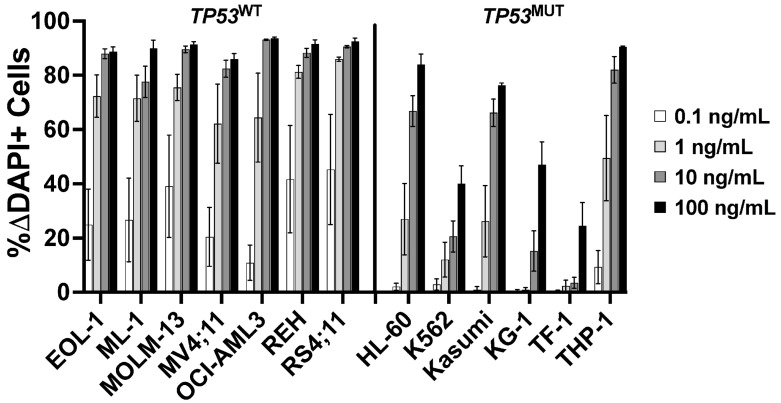
Association between *TP53* mutational status and CLM-induced cytotoxicity. Parental cell lines were treated with various doses of CLM. After 3 days, drug-induced cytotoxicity was assessed flow cytometrically. Data are presented as mean ± SEM from 4 independent experiments performed in duplicate wells.

**Figure 3:**
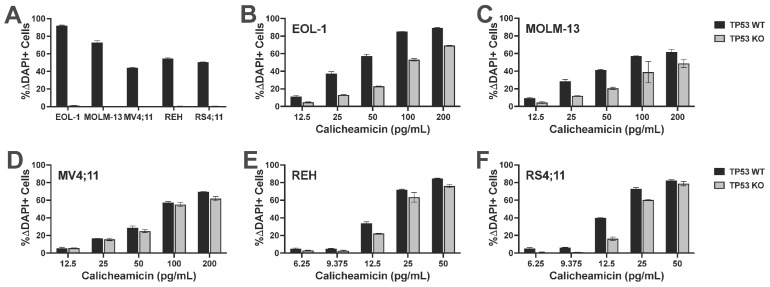
*TP53* deletion leads to relative CLM resistance in syngeneic acute leukemia cell line models. *TP53*^WT^ cell lines were electroporated with *TP53*-targeting Cas9/CRISPR sgRNA nucleoprotein complexes and bulk cells were then treated with 1.0 or 2.5 µM idasanutlin for 14 days to enrich for *TP53*^KO^ cells. (**A**) Parental cells electroporated with Cas9 alone and idasanutlin-enriched *TP53*^KO^ cells were treated with 1.0 or 2.5 µM idasanutlin, and idasanutlin-induced cytotoxicity was assessed flow cytometrically after 3 days. *TP53*^WT^ and idasanutlin-enriched *TP53*^KO^ (**B**) EOL-1, (**C**) MOLM-13, (**D**) MV4;11, (**E**) REH, and (**F**) RS4;11 cell pairs were then subjected to various concentrations of CLM for 3 days before cell numbers and viability were assessed. Data are shown as percent change in DAPI-positive cells and are presented as mean ± SD. Shown is one representative of three qualitatively similar, independent experiments performed in duplicate wells. A second series of experiments with an independent electroporation yielded qualitatively similar results.

Having identified *TP53* as a possible CLM sensitivity gene in our screening, we evaluated the association between *TP53* mutations and CLM-induced cytotoxicity in a set of 13 human leukemic cell lines. This set included seven *TP53* wild-type (*TP53*^WT^: EOL-1, ML-1, MOLM-13, MV4;11, OCI-AML3, REH, and RS4;11) and six *TP53* mutant (*TP53*^MUT^; HL-60, K562, Kasumi, KG-1, TF-1, and THP-1) cell lines we identified via DNA sequencing for all coding exons of *TP53* [18]. Across these 13 acute leukemia cell lines, the 6 *TP53*^MUT^ cell lines were approximately 10- to 1000-fold less sensitive to CLM than the 7 *TP53*^WT^ cell lines. For example, at 1 ng/mL, the median (±SEM) CLM-induced cell death was 19 ± 8% in *TP53*^MUT^ cells and 72 ± 3% in *TP53*^WT^ cells (Figure 2). To test the relationship between *TP53* and CLM sensitivity further, we then derived *TP53*^WT/KO^ syngeneic cell line pairs via deletion of *TP53* from bulk cells via CRISPR/Cas9 and exposure to 1–2.5 µM of the mouse double minute 2 (MDM2) inhibitor, idasanutlin, to enrich for the population of *TP53*^KO^ cells [18]. As shown in Figure 3, *TP53*^KO^ sublines indeed showed reduced sensitivity to their *TP53*^WT^ counterparts in all five cell line models, although the degree of relative resistance varied significantly across cell lines.

### 3.3. Validation of Role of DNA Damage Pathways in CLM Sensitivity

**Figure 4:**
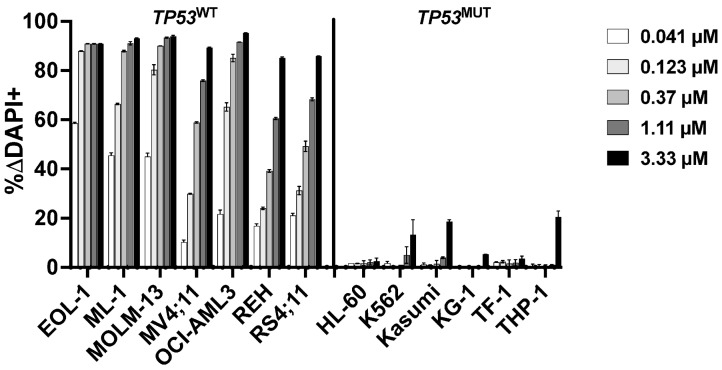
Association between *TP53* mutational status and idasanutlin-induced cytotoxicity. Idasanutlin-induced cytotoxicity was assessed flow cytometrically after 3 days. Data are presented as mean ± SD from one representative experiment performed in duplicate wells.

**Figure 5:**
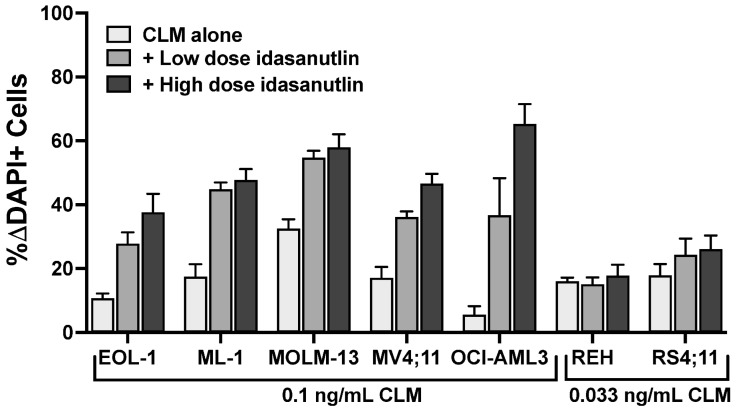
Idasanutlin enhances CLM-induced cytotoxicity in *TP53*^WT^ leukemia cell lines. Parental *TP53*^WT^ leukemia cell lines were treated with a sub-maximally effective dose of CLM in the absence or presence of either a low or high dose of idasanutlin (5 or 10 nM for EOL-1 and MOLM-13 cells; 10 or 20 nM for ML-1, MV4;11, OCI-AML3, REH, and RS4;11 cells). After 3 days, cell numbers and the percentage of dead cells were quantified by flow cytometry. Data are shown as percent change in DAPI-positive cells relative to idasanutlin/medium alone and are presented as mean ± SEM from 3 independent experiments performed in duplicate wells.

Because *TP53* and *CHEK2* are in the same cellular DNA damage response pathway as *MDM2* and ataxia–telangiectasia mutated (*ATM*) [19,20,21], we next tested which inhibitors of this pathway regulated response to CLM. We first tested the mouse double minute 2 (MDM2) inhibitor idasanutlin, a strong activator of p53. Nanomolar concentrations of idasanutlin effectively killed parental *TP53*^WT^ leukemia cell lines. By comparison, much higher doses (3–10 µM) were required to kill parental leukemia cell lines harboring *TP53* alterations, in line with the notion that *TP53* inactivation render cells insensitive to MDM2 inhibition [22] (Figure 4). We therefore assessed whether idasanutlin enhanced CLM-induced cytotoxicity in *TP53*^WT^ cells. Indeed, across the seven *TP53*^WT^ cell lines, drug-induced toxicity increased from a median of 7 ± 5% ΔDAPI+ cells (CLM alone at 0.05 ng/mL) to 31 ± 3% ΔDAPI+ cells when idasanutlin (10–20 nM) was added (Figure 5). In contrast, *TP53*^MUT^ cells were resistant (<5% ΔDAPI+ cells) to idasanutlin even at 1 µM, and no combinatory effect was seen with CLM (Supplementary Figure S1).

**Figure 6:**
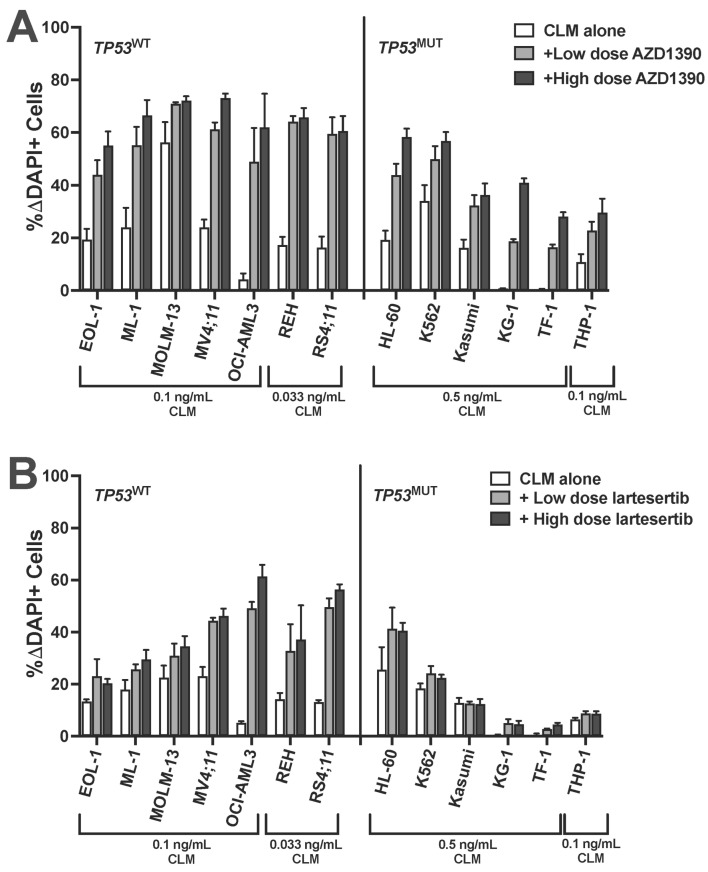
ATM inhibitors enhance CLM-induced cytotoxicity in acute leukemia cell lines independent of *TP53* status. A panel of human acute leukemia cell lines was treated with a sub-maximally effective dose of CLM in the absence or presence of either a low or high dose of (**A**) AZD1390 (0.1 and 0.25 µM: REH; 0.25 and 1 µM: EOL-1, MV4;11, OCI-AML3, RS4;11, Kasumi, and KG-1; 0.25 and 2.5 µM: MOLM-13, THP-1, and HL-60; 1 and 2.5 µM: TF-1; 2.5 and 5 µM: ML-1 and K562) or (**B**) lartesertib (1 and 2.5 µM: EOL-1, MOLM-13, MV4;11, REH, RS4;11, HL-60, Kasumi, and KG-1; 2.5 and 5 µM: ML-1, OCI-AML3, K562, TF-1, and THP-1). After 3 days, and cell numbers and the percentage of dead cells were quantified by flow cytometry. Data are shown as percent change in DAPI-positive cells relative to AZD1390 or lartesertib/medium alone and are presented as mean ± SEM from 3 independent experiments performed in duplicate wells.

We then tested small-molecule inhibitors that could activate DNA damage pathways upstream of *TP53*. One such pharmacological target is ATM. Treatment of cells with the ATM inhibitor AZD1390 has been shown to cause cell death in *TP53*^MUT^ cells [23]. At relatively non-toxic doses (Supplementary Figure S2), AZD1390 enhanced CLM-induced cytotoxicity in all 13 tested human leukemia cell lines, including all 6 *TP53*^MUT^ cell lines (Figure 6A). Specifically, in *TP53*^WT^ cell lines, median drug-induced cell death increased from 7 ± 6% with 0.05 ng/mL CLM alone to 41 ± 9% with the addition of 0.25–2.5 µM of AZD1390; across *TP53*^MUT^ cell lines, the median drug-induced cell death increased from 19 ± 13% with 0.5 ng/mL of CLM to 50 ± 10% with the addition of 1–2.5 µM of AZD1390. Testing of a second ATM inhibitor, lartesertib, yielded qualitatively similar results, except for one cell line (Kasumi) which failed to show any evidence of sensitivity to lartesertib (Figure 6B and Supplementary Figure S3). In contrast, no significant or consistent effects on CLM-induced cytotoxicity were found in combination treatments with an ATR inhibitor (ceralasertib; Supplementary Figure S4), a Chk1/Chk2 inhibitor (prexasertib; Supplementary Figure S5), a Chk2 inhibitor (BML-277; Supplementary Figure S6), or a PARP inhibitor, veliparib. With the latter, for example, in *TP53*^WT^ cell lines, median drug-induced cell death changed from 3 ± 3% with 0.05 ng/mL CLM alone to 7 ± 6% with the addition of 1–10 µM of veliparib across *TP53*^WT^ cell lines. Across the leukemia cell lines with *TP53* alteration, the median drug-induced cell death changed from 5 ± 5% with 0.5 ng/mL of CLM to 7 ± 7% with the addition of 2.5–10 µM of veliparib (Supplementary Figure S7).

## 4. Discussion

With GO and InO, CLM-based ADCs are routinely employed in patients with acute leukemia nowadays. However, while they are effective in subsets of patients with CD33+ AML or CD22+ B-ALL, respectively [7,8,9,10,11,12], others do not meaningfully benefit from these drugs as they are currently used. To identify genetic vulnerabilities and new biomarkers that could inform the selection of patients most likely to benefit from CLM-based ADCs and to develop rational combination therapies that augment the efficacy of these ADCs, we performed genome-wide CRISPR/Cas9 screening to discover genes associated with CLM sensitivity. In this screen, human acute leukemia cell lines with genetic lesions introduced by CRISPR/Cas9 were exposed to CLM and surviving cells were analyzed for over- or under-representation of genetic lesions relative to the starting cell population as a read-out for resistance or sensitivity to CLM, respectively.

As a main finding, our screen identified *TP53* as a key sensitivity gene for CLM, with genetic *TP53* deletion being the top hit and leading to relative CLM resistance in all three acute leukemia cell backgrounds tested, including K562 cells which harbor a gain-of-function *TP53* point mutation (Q136P) [24] that was possibly selected against in our screening. Consistent with these results, we found human leukemia cell lines with *TP53* alterations to be 10- to 1000-fold less sensitive to CLM than *TP53*^WT^ cell lines across the panel of 13 cell lines we studied. To study the impact of *TP53* on CLM sensitivity more directly, we also derived *TP53*^KO^ sublines from five *TP53*^WT^ cell lines. In these studies, *TP53*^KO^ cells were not completely resistant to CLM but were significantly less sensitive to CLM than their *TP53*^WT^ counterparts, further demonstrating the impact *TP53* has on CLM-induced cytotoxicity. Together, these data point to the acute leukemia cells’ *TP53* status—a key marker for the sensitivity of both AML and B-ALL to conventional chemotherapy [25,26,27,28]—as a biomarker for patient selection or, in clinical drug testing, patient stratification when CLM-based ADCs are used. Whether *TP53*-mutated leukemia cells’ sensitivity to CLM-based ADCs is affected by co-mutations that may be present is an open question in need of further study.

Having identified *TP53* as a key sensitivity gene for CLM, we then focused our efforts on genes in DNA damage pathways that are affected by *TP53*, in particular *MDM2* and *ATM* [19,20,21]. In *TP53*^WT^ but not *TP53*^MUT^ cells, the MDM2 inhibitor and p53 activator, idasanutlin, enhanced CLM cytotoxicity, demonstrating that decoupling of cells from MDM2-p53 regulation sensitizes leukemia cells to CLM. Moreover, both ATM inhibitors we tested, AZD1390 and lartesertib, significantly enhanced CLM efficacy. Consistent with our data, previous investigations with SV40-transformed fibroblasts derived from ataxia telangiectasia (AT) patients showed hypersensitivity to a calicheamicin derivative [29]. Of potential clinical interest, the effect of ATM inhibitors was independent of the *TP53* status, unlike the effect of the MDM2 inhibitor. In contrast to reports by others [30,31], a PARP inhibitor (veliparib) only minimally affected CLM-induced cytotoxicity in our panel of human acute leukemia cell lines. Likewise, CLM-induced cytotoxicity was not significantly modulated by an ATR inhibitor, a Chk1/Chk2 inhibitor, or a Chk2 inhibitor. The latter was unexpected considering that the Chk2-p53 pathway is well established and both TP53 and CHEK2 were identified in our screen and may suggest a role of residual non-catalytic functions of Chk2 in the leukemia cells’ response to CLM-induced cytotoxicity.

Our observation that inhibition of ATM or MDM2 sensitizes acute leukemia cells to CLM may provide the rationale for the clinical exploration of a combination therapy with either GO or InO. While efficacy has been limited thus far, several MDM2 inhibitors have been tested in patients with acute leukemia [32,33,34,35]. Testing of ATM inhibitors in acute leukemia is so far largely confined to preclinical investigations but early-phase clinical trials in other cancers have been completed [36] or initiated (e.g., NCT03423628, NCT05116254, NCT05182905, NCT05396833, NCT06433219, NCT06894979). Our data would support the testing of an MDM2 inhibitor with either GO or InO in patients with *TP53*^WT^ acute leukemia, whereas the combination of GO or InO with an ATM inhibitor could be explored in patients independent of the *TP53* mutational status. It is interesting to speculate—and a goal of future studies—whether p53 reactivators like eprenetapopt (APR-246) [37] might be of value to sensitize acute leukemias with TP53 mutations to CLM-based ADCs.

In our studies, we exclusively focused on CLM-containing ADCs. While our findings do not immediately apply to ADCs delivering other small-molecule toxins, as resistance mechanisms may differ, our experimental approach may offer a blueprint for the identification of genetic vulnerabilities that are critical for the anti-tumor activity of other ADCs.

## 5. Conclusions

Our study identified *ATM*, *MDM2*, and *TP53*—which exhibit the same cellular response to DNA damage pathways—as key modulators of CLM-induced cytotoxicity in acute leukemia cells. While these findings may not be surprising per se, considering the mechanism of action of CLM [8,13], the observation that our genomic screening identified these modulators as key hits may suggest their potential value as targets for combination therapies. Thus, our results support further evaluation of combination therapies with corresponding small-molecule inhibitors targeting MDM2 or ATM toward clinical testing as novel strategies to increase the efficacy of CLM-based ADCs such as GO and InO.

## Data Availability

For original data and reagents, please contact the corresponding author (rwalter@fredhutch.org).

## Supplementary Materials

The following supporting information can be downloaded at https://www.mdpi.com/article/10.3390/cancers18010067/s1, Figure S1: Effect of idasanutlin on CLM-induced cytotoxicity in *TP53*^MUT^ leukemia cell lines. Figure S2: AZD1390-induced cytotoxicity in acute leukemia cell lines. Figure S3: Lartesertib-induced cytotoxicity in acute leukemia cell lines. Figure S4: Effect of ceralasertib on CLM-induced cytotoxicity in human acute leukemia cell lines. Figure S5: Effect of prexasertib on CLM-induced cytotoxicity in human acute leukemia cell lines. Figure S6: Effect of BML-277 on CLM-induced cytotoxicity in human acute leukemia cell lines. Figure S7: Effect of veliparib on CLM-induced cytotoxicity in human acute leukemia cell lines.
