# Supplementary material for: DNA Damage Sensing and TP53 Function as Modulators of Sensitivity to Calicheamicin-Based Antibody–Drug Conjugates for Acute Leukemia
Source: Cancers (Basel). 2025 Dec 25;18(1):67. doi: 10.3390/cancers18010067 (PMC12784841; doi:10.3390/cancers18010067)
Supplement: Supplementary file 1 [file cancers-18-00067-s001.zip › cancers-4038024-supplementary.pdf]

SUPPLEMENTARY FIGURE S1

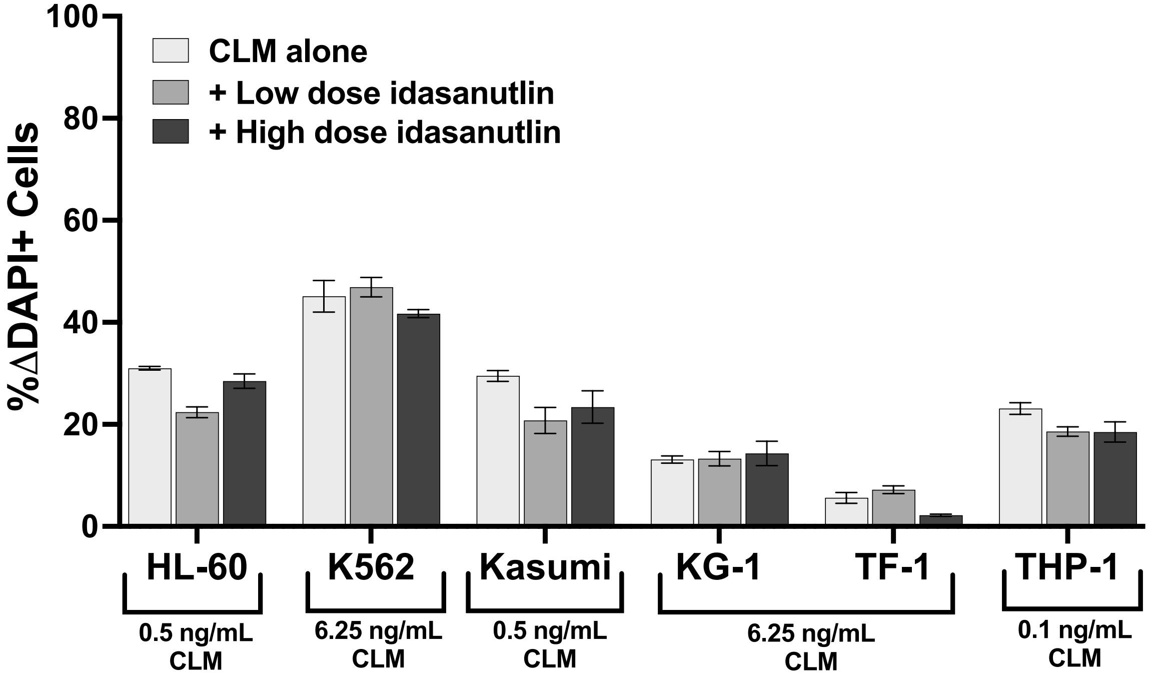

Effect of idasanutlin on CLM-induced cytotoxicity in *TP53*^{MUT} leukemia cell lines. Parental *TP53*^{MUT} leukemia cell lines were treated with a sub-maximally effective dose of CLM in the absence or presence of either a lower (0.5 µM) or higher (1 µM) dose of idasanutlin. After 3 days, cell numbers and the percentage of dead cells were quantified by flow cytometry. Data are shown as percent change in DAPI-positive cells relative to idasanutlin/medium alone and are presented as mean ± SD from one experiment performed in duplicate wells.

SUPPLEMENTARY FIGURE S2

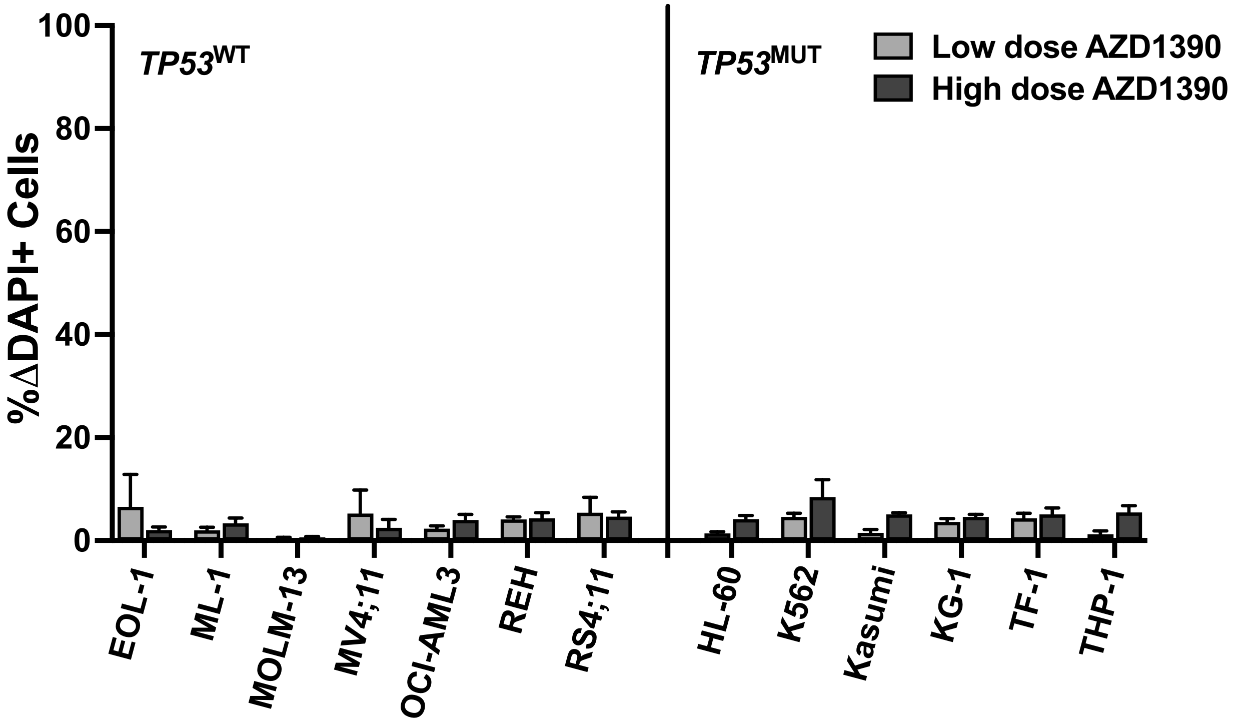

AZD1390-induced cytotoxicity in acute leukemia cell lines. A panel of human acute leukemia cell lines was treated with AZD1390 (0.1 and 0.25 µM: REH; 0.25 and 1 µM: EOL-1, MV4;11, OCI-AML3, RS4;11, Kasumi, and KG-1; 0.25 and 2.5 µM: MOLM-13, THP-1, and HL-60; 1 and 2.5 µM: TF-1; 2.5 and 5 µM: ML-1 and K562). After 3 days, cell numbers and the percentage of dead cells were quantified by flow cytometry. Data are shown as percent change in DAPI-positive cells relative to cells treated without AZD1390 and are presented as mean±SEM from 3 independent experiments performed in duplicate wells.

SUPPLEMENTARY FIGURE S3

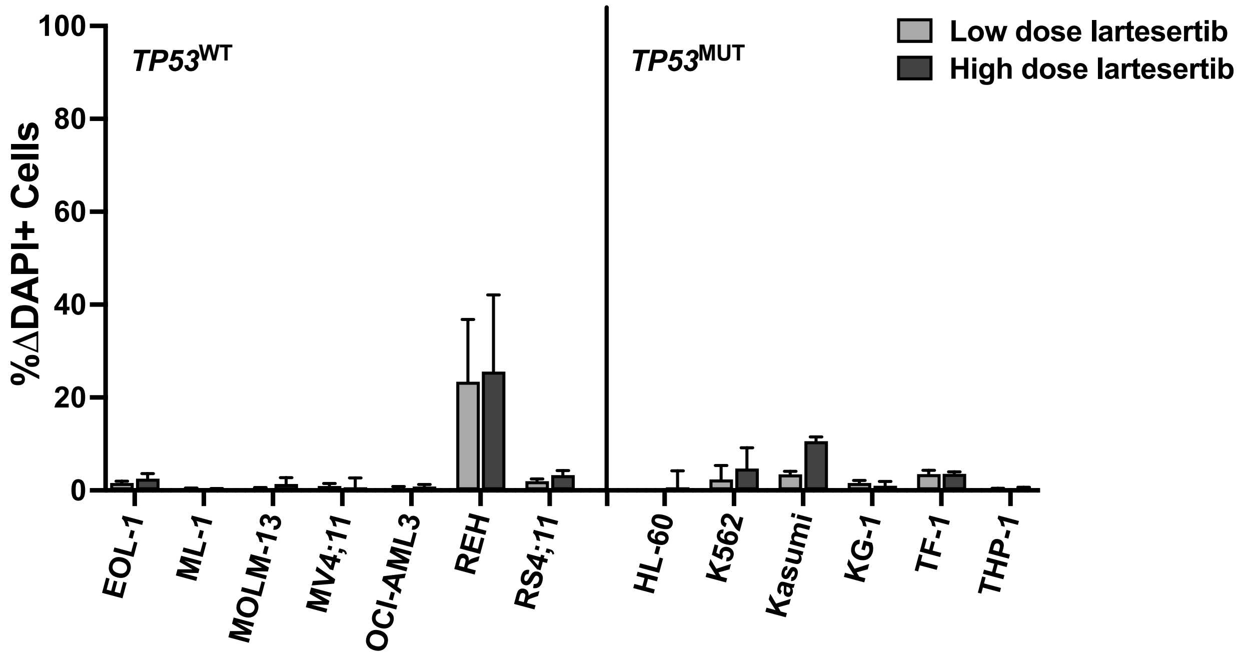

Lartesertib-induced cytotoxicity in acute leukemia cell lines. A panel of human acute leukemia cell lines was treated with lartesertib (1 and 2.5 μM: EOL-1, MOLM-13, MV4;11, REH, RS4;11, HL-60, Kasumi, and KG-1; 2.5 and 5 μM: ML-1, OCI-AML3, K562, TF-1, and THP-1). After 3 days, cell numbers and the percentage of dead cells were quantified by flow cytometry. Data are shown as percent change in DAPI-positive cells relative to cells treated without lartesertib and are presented as mean±SEM from 3 independent experiments performed in duplicate wells.

SUPPLEMENTARY FIGURE S4

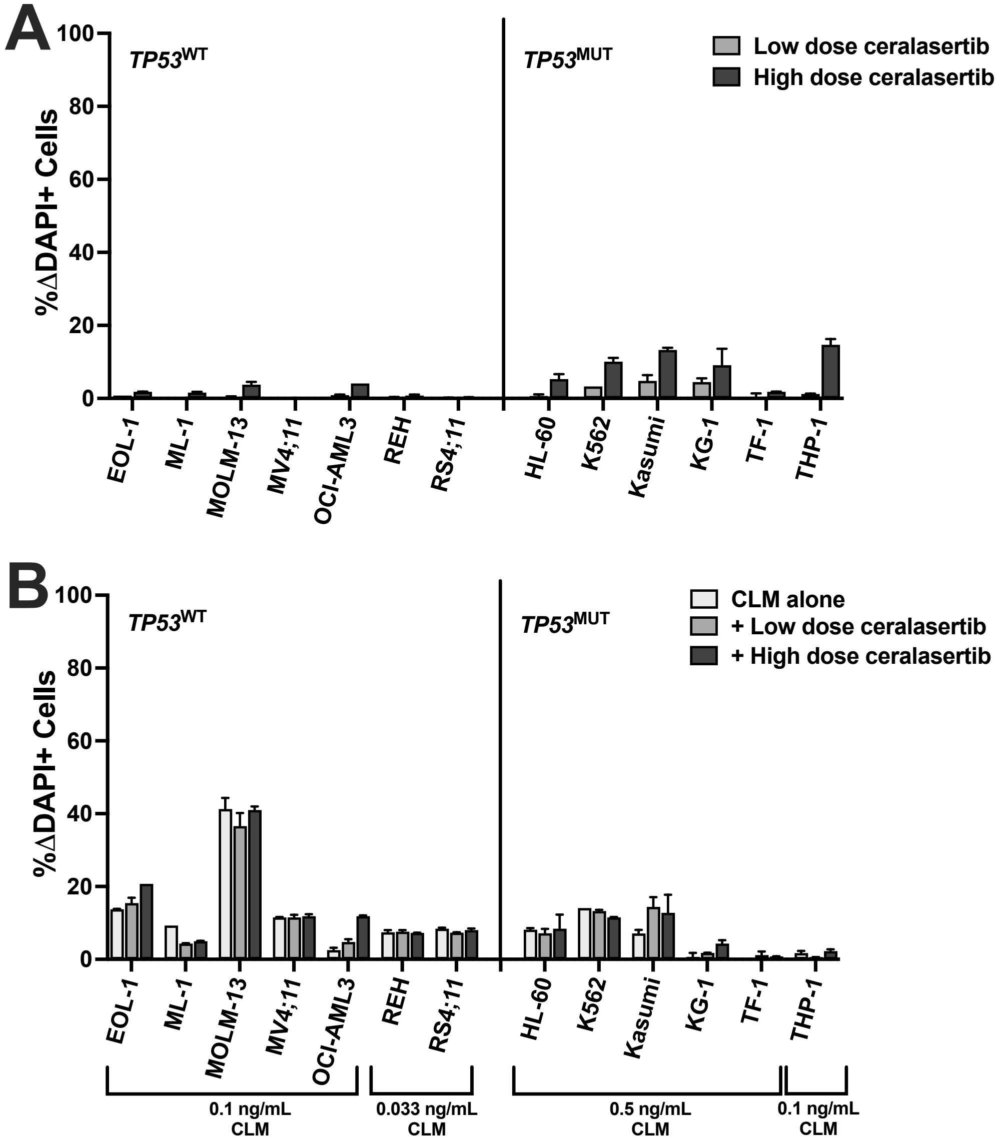

Effect of ceralasertib on CLM-induced cytotoxicity in human acute leukemia cell lines. (A) A panel of human acute leukemia cell lines was treated with ceralasertib (0.125-4 µM) or left untreated. **(B)** Cell lines were treated with a sub-maximally effective dose of CLM in the absence or presence of either a low or high dose of ceralasertib (0.125 and 0.25 µM: EOL-1, REH, and RS4;11; 0.5 and 0.75 µM: ML-1, MOLM-13, MV4;11, OCI-AML3, and TF-1; 0.75 and 2.5 µM: THP-1; 2.5 and 4 µM: HL-60, K562, Kasumi, and KG-1). Cell numbers and the percentage of dead cells were quantified by flow cytometry. Data are shown as percent change in DAPI-positive cells relative to cells treated without ceralasertib and are presented as mean±SD from one representative out of two experiments performed in duplicate wells.

SUPPLEMENTARY FIGURE S5

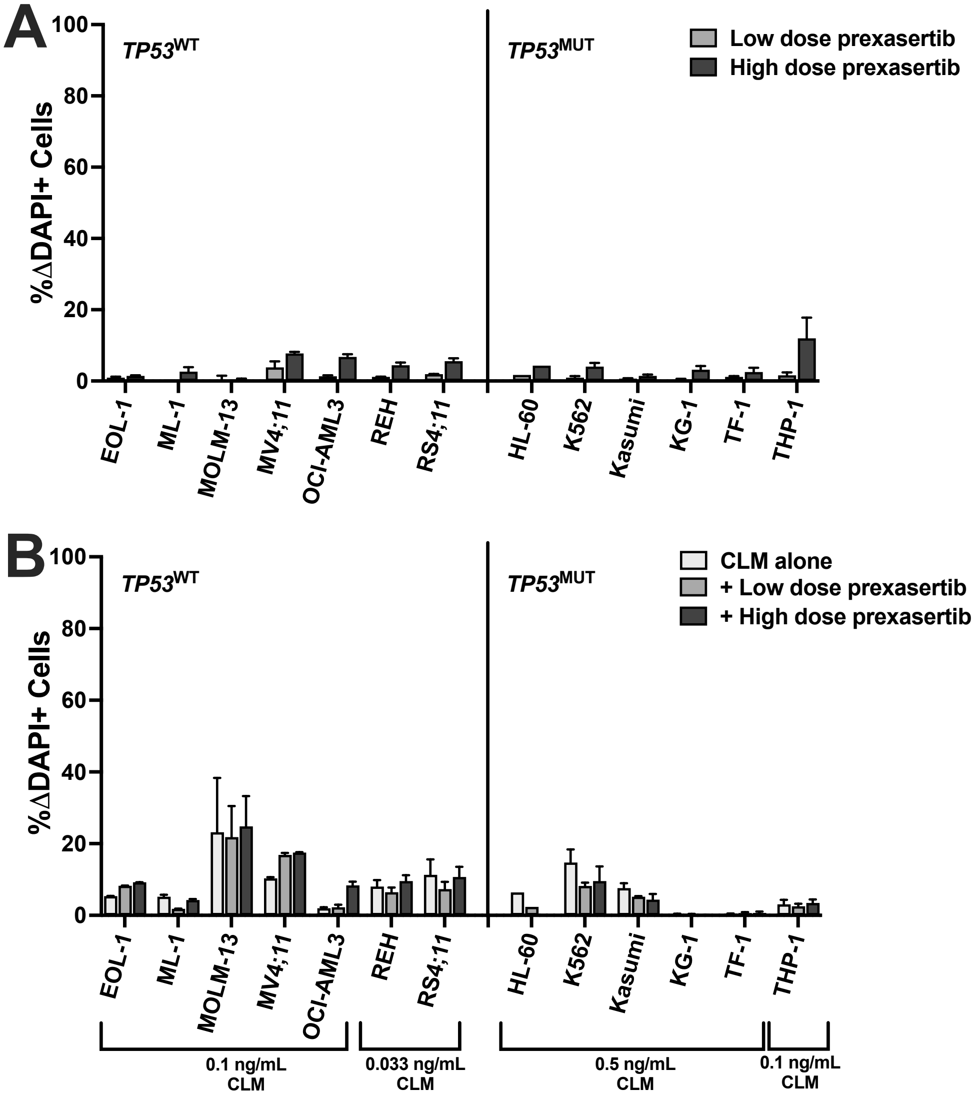

Effect of prexasertib on CLM-induced cytotoxicity in human acute leukemia cell lines. (A) A panel of human acute leukemia cell lines was treated with prexasertib (0.5-200 nM) or left untreated. **(B)** Cell lines were treated with a sub-maximally effective dose of CLM in the absence or presence of either a low or high dose of prexasertib (0.5 and 1 nM: EOL-1, MOLM-13, MV4;11, and Kasumi; 25 and 50 nM: ML-1 and HL-60; 50 and 100: OCI-AML3, REH, RS4:11, K562, TF-1, and THP-1; 100 and 200: KG-1). Cell numbers and the percentage of dead cells were quantified by flow cytometry. Data are shown as percent change in DAPI-positive cells relative to cells treated without prexasertib and are presented as mean±SEM from two independent experiments performed in duplicate wells (one independent experiment performed in duplicate wells for HL-60).

SUPPLEMENTARY FIGURE S6

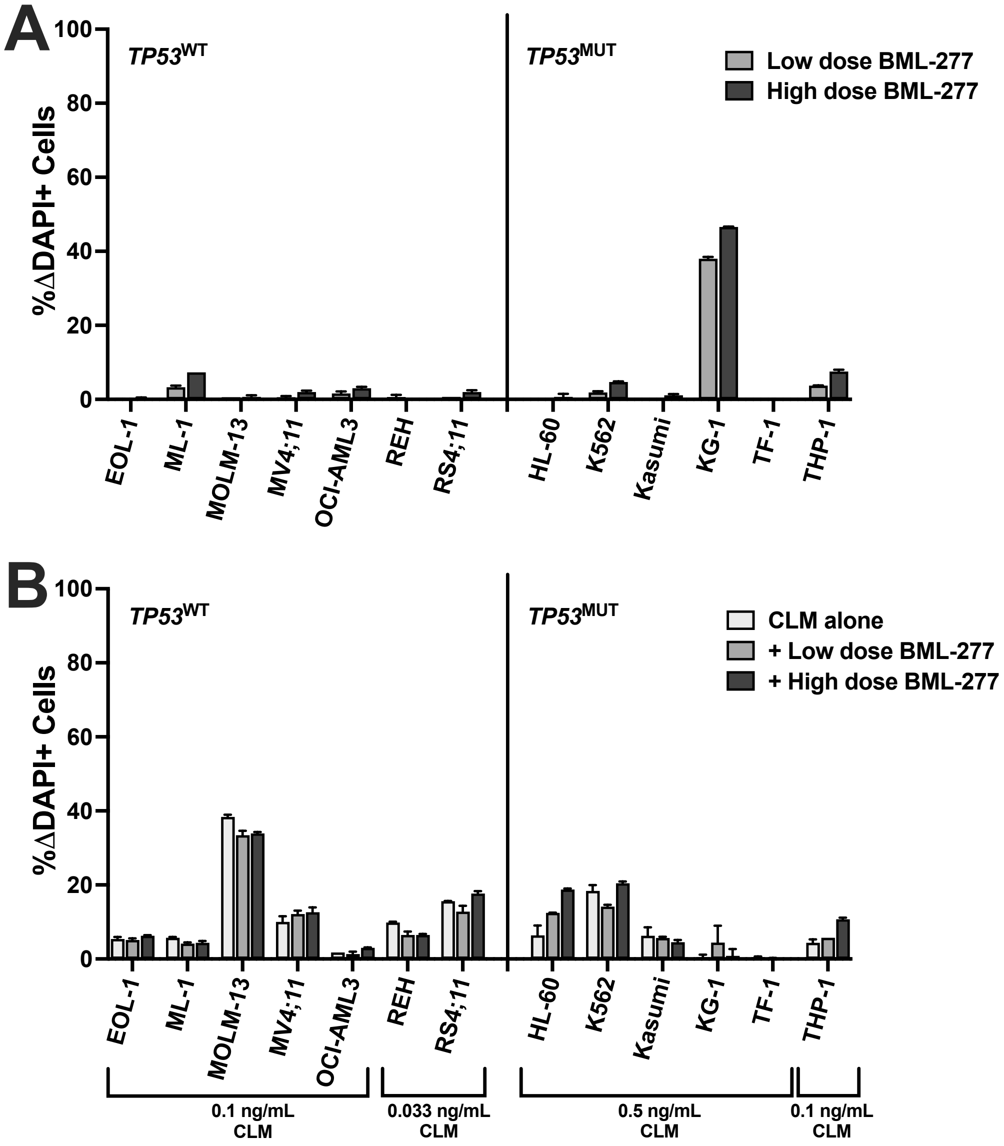

Effect of BML-277 on CLM-induced cytotoxicity in human acute leukemia cell lines. (A) A panel of human acute leukemia cell lines was treated with BML-277 (0.625-10 µM) or left untreated. **(B)** Cell lines were treated with a sub-maximally effective dose of CLM in the absence or presence of either a low or high dose of BML-277 (0.625 and 1.25 µM: EOL-1, MOLM-13, MV4;11, and KG-1; 1.25 and 2.5 µM: OCI-AML3, REH, and Kasumi; 2.5 and 5 µM: K562, TF-1, and THP-1; 5 and 10: ML-1, RS4;11, and HL-60). Cell numbers and the percentage of dead cells were quantified by flow cytometry. Data are shown as percent change in DAPI-positive cells relative to cells treated without BML-277 and are presented as mean±SD from one representative out of two experiments performed in duplicate wells.

SUPPLEMENTARY FIGURE S7

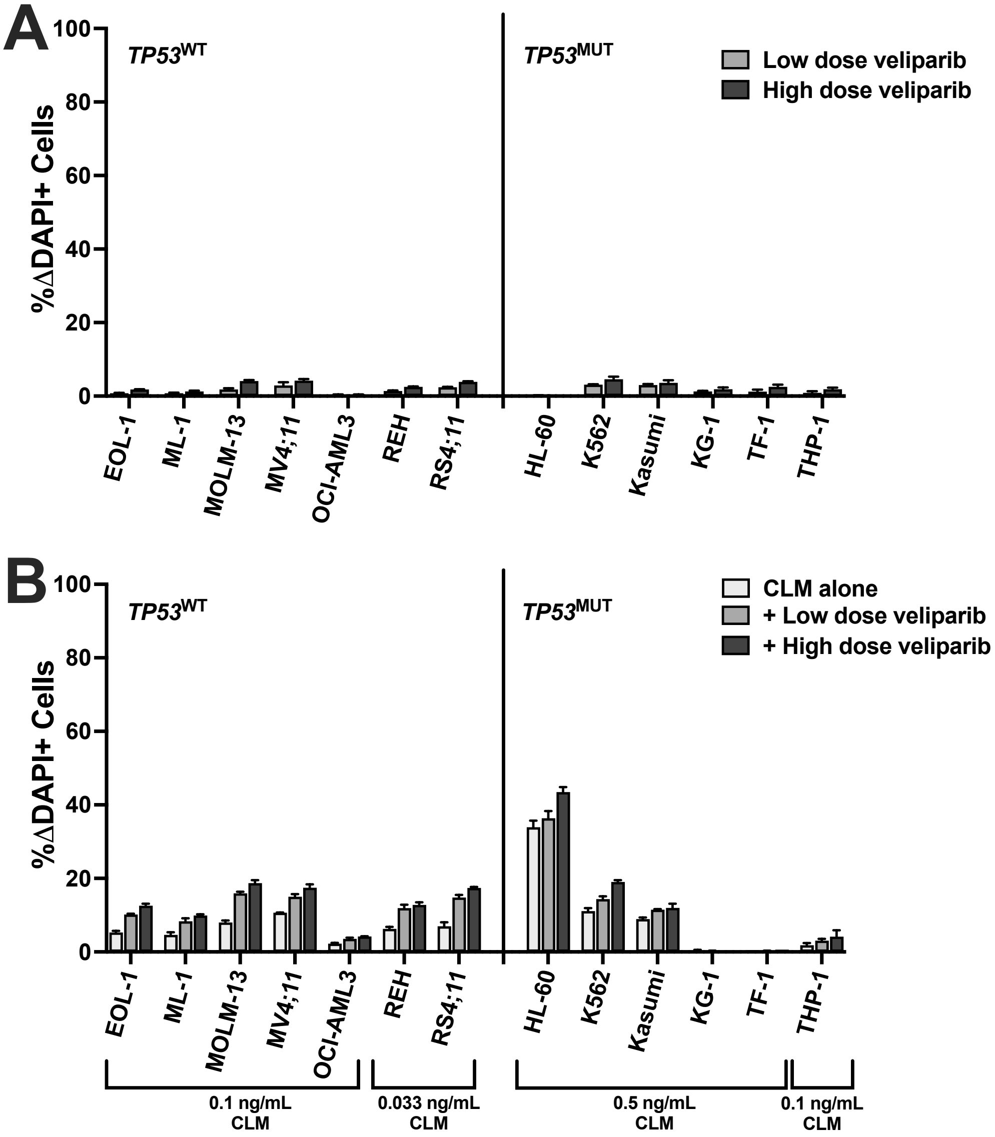

Effect of veliparib on CLM-induced cytotoxicity in human acute leukemia cell lines. (A) A panel of human acute leukemia cell lines was treated with veliparib (1-10 μM) or left untreated. **(B)** Cell lines were treated with a sub-maximally effective dose of CLM in the absence or presence of either a low or high dose of veliparib (1 and 2.5 μM: OCI-AML3, REH, and RS4;11; 2.5 and 5 μM: ML-1, MV4;11, Kasumi, KG-1, and TF-1; 5 and 10 μM: EOL-1, MOLM-13, HL-60, K562, and THP-1). Cell numbers and the percentage of dead cells were quantified by flow cytometry. Data are shown as percent change in DAPI-positive cells relative to cells treated without veliparib and are presented as mean±SD from one representative out of two experiments performed in duplicate wells.
